# Integrative analysis of genomic, epigenomic and transcriptomic data identified molecular subtypes of esophageal carcinoma

**DOI:** 10.18632/aging.202556

**Published:** 2021-02-26

**Authors:** Mingyang Ma, Yang Chen, Xiaoyi Chong, Fangli Jiang, Jing Gao, Lin Shen, Cheng Zhang

**Affiliations:** 1 Department of Gastrointestinal Oncology, Key Laboratory of Carcinogenesis and Translational Research (Ministry of Education/Beijing), Peking University Cancer Hospital and Institute, Beijing 100142, China; 2 National Cancer Center/National Clinical Research Center for Cancer/Cancer Hospital and Shenzhen Hospital, Chinese Academy of Medical Sciences and Peking Union Medical College, Shenzhen 518116, China

**Keywords:** esophageal cancer, prognostic markers, copy number variation, methylation, multi-omics associated analysis

## Abstract

Esophageal cancer (EC) involves many genomic, epigenetic and transcriptomic disorders, which play key roles in the heterogeneous progression of cancer. However, the study of EC with multi-omics has not been conducted. This study identified a high consistency between DNA copy number variations and abnormal methylations in EC by analyzing genomics, epigenetics and transcriptomics data and investigating mutual correlations of DNA copy number variation, methylation and gene expressions, and stratified copy number variation genes (CNV-Gs) and methylation genes (MET-Gs). The methylation, CNVs and expression profiles of CNV-Gs and MET-Gs were analyzed by consistent clustering using iCluster integration, here, we determined three subtypes (iC1, iC2, iC3) with different molecular traits, prognostic characteristics and tumor immune microenvironment features. We also identified 4 prognostic genes (CLDN3, FAM221A, GDF15 and YBX2) differentially expressed in the three subtypes, and could therefore be used as representative biomarkers for the three subtypes of EC. In conclusion, by performing comprehensive analysis on genomic, epigenetic and transcriptomic regulations, the current study provided new insights into the multilayer molecular and pathological traits of EC, and contributed to the precision medication for EC patients.

## INTRODUCTION

Esophageal carcinoma (EC), which is one of the most aggressive types of cancers, has now become the sixth leading cause of cancer-related death all over the world [1]. The vast majority of EC take place at the upper and middle esophagus and are histologically classified as esophageal squamous cell carcinoma (ESCC), while those cases occurring at the lower esophagus near the stomach junction are classified as esophageal adenocarcinoma (EAC) [2, 3]. China accounts for 70% of all EC cases, which are predominantly composed of ESCC subtypes [2, 4, 5]. More than half of EC patients have already with distant metastases at diagnosis and tend to develop a 5-year survival of between 10% and 20% [1]. Therefore, it is urgent to determine effective prognostic biomarkers from multiple perspectives to facilitate a more accurate prediction of clinical outcome and provide references for targeted drug development against EC.

With the advent of new biochemical technologies (especially next-generation sequencing), cancer genomic characteristics could be systematically analyzed. Recently, the dysregulation in cancers has been widely investigated at genomic levels by performing large-scale multi-omics analysis [6]. Genomic variation as a result of DNA copy number variation (CNV) and single nucleotide mutations (SNPs) could easily lead to tumor development [7, 8]. DNA copy number variation played a key regulatory role in the progression of ESCC [9, 10], and transcriptional disorders caused by copy number changes were potential driving events in EC progression [11]. On the other hand, analysis of DNA methylation profiles has demonstrated the vast heterogeneity of epigenome disorders in EC and other cancer types [12–14], and further studies also proved that DNA methylation contributes to heterogeneous biological behaviors and is actively involved in the progression of ESCC [15–17]. These open, large-scale, multi-omics data sets make it possible for conducting a comprehensive multi-omics analysis based on genomics, epigenomics and transcriptomics to improve the prognostic prediction of EC.

**Figure 1:**
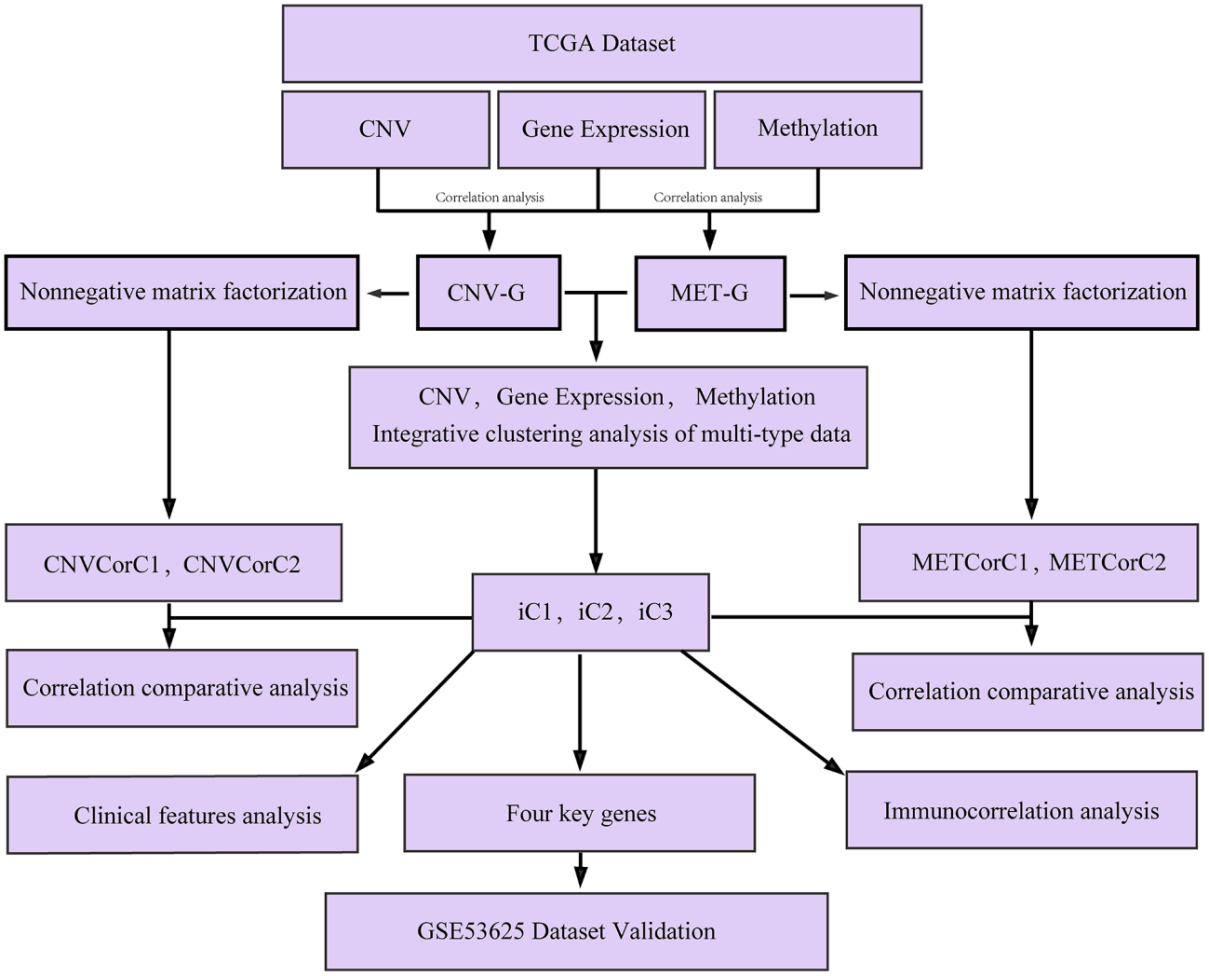
**Work flow chart.**

There may be co-regulations between DNA copy number and DNA methylation abnormalities, as both the two have been found to exert important effects on EC development [18, 19]. However, their potential relationship in EC development has not been well studied. In this study, by performing multi-omics integration, we analyzed gene expressions dysregulated by genomic or epigenetic modes, and identified different molecular subtypes significantly associated with EC prognosis, the work flow chart is shown in Figure 1. This study identified novel subtypes and biomarkers for precision medicine and provided a basis for better understanding of the molecular mechanisms of EC development and progression.

## RESULTS

### DNA copy number abnormalities were highly consistent with methylation abnormalities

**Figure 2:**
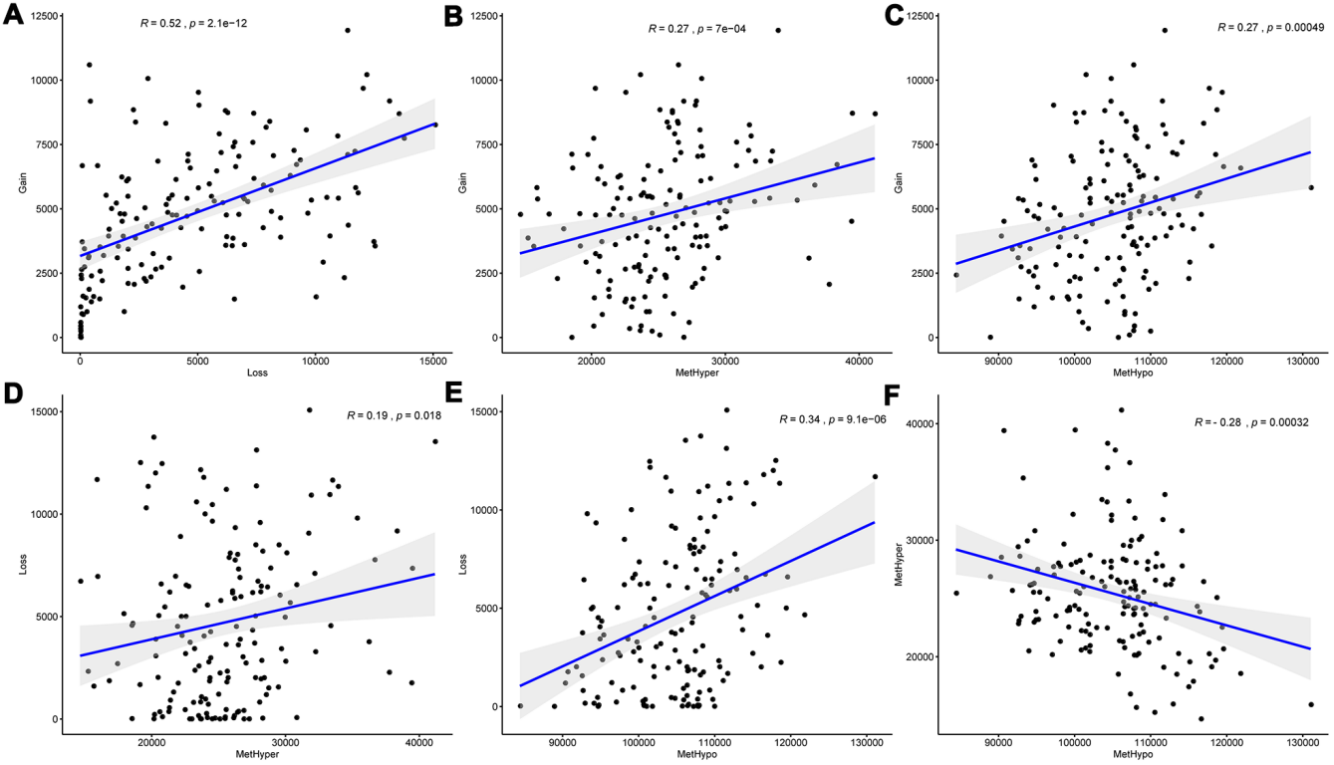
**DNA copy number anomalies were highly consistent with methylation abnormalities.** (**A**) Correlation between frequencies of CNV Gain and Loss. (**B**) Correlation between frequencies of CNV Gain and MetHyper. (**C**) Correlation between frequencies of CNV Gain and MetHypo. (**D**) Correlation between frequencies of CNV Loss and MetHyper. (**E**) Correlation between frequencies of CNV Loss and MetHypo. (**F**) Correlation between frequencies of MetHyper and MetHypo. Correlation was calculated using the Pearson correlation coefficient.

DNA copy number and DNA methylation abnormalities have an important impact on the progression of EC. To examine the relationship between the two, we defined the CNV value of CNV > 0.3 as gain, < -0.3 as Loss, the β value of methylation > 0.8 as hypermethylation (MetHyper) and < 0.2 as demethylation (MetHypo). The number of CNV Gain, CNV Loss, MetHyper and MetHypo for each sample were counted to analyze the relationship between the frequency of CNV Gain and CNV Loss in each sample, and we detected a significant positive correlation (R=0.52, p=2.1e-12) (Figure 2A), which suggested that high frequency of copy number amplification events in the EC patients’ genome were accompanied by high frequency of deletions. Similarly, the frequency of CNV Gain in each patient was significantly positively correlated with the frequency of MetHyper (R=0.27, p=7e-04) (Figure 2B), and the frequency of CNV Gain in each patient also showed a close correlation with the frequency of MetHypo (R=0.27, p=0.00049) (Figure 2C). Moreover, the frequency of CNV Loss was significantly positively correlated with the frequency of MetHyper in each patient (R=0.19, p=0.0018) (Figure 2D), and a significant positive correlation between the frequency of CNV Loss and the frequency of MetHypo was detected in each patient (R=0.34, p=9.1e-06) (Figure 2E). These results indicated that EC patients’ genome instability was accompanied by abnormal DNA methylation. Furthermore, the occurrence of MetHyper frequency and MetHypo in each patient was determined to be closely negatively correlated (R=-0.28, P =0.00032) (Figure 2F). The occurrence of DNA hypermethylation and hypomethylation events in patients seemed to be mutually exclusive. These results suggested that patients with frequent CNV dysregulation was more likely to exhibit methylation disorders, and that DNA copy number abnormalities and methylation abnormalities might be co-regulatory.

### Identification of CNV-G and MET-G gene sets

**Figure 3:**
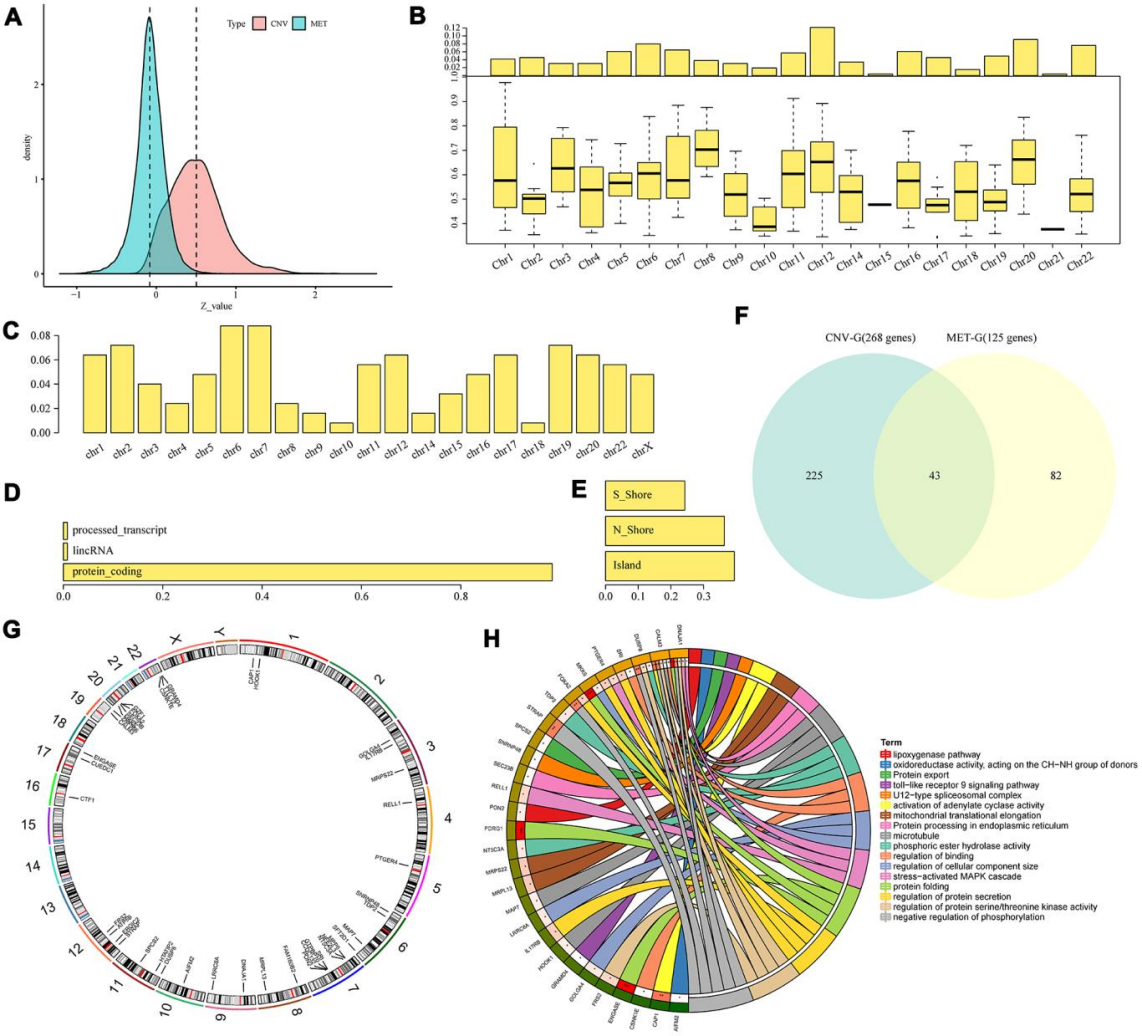
**Identification of CNV-G and MET-G gene sets.** (**A**) Correlation z-values between CNV (CNV-G) and expression profiles, or between methylation (MET-G) and expression profiles. Distributions of (**B**) CNV-Gs and (**C**) MET-Gs on the genome were mapped. (**D**) Functional composition and (**E**) distribution of methylation sites were determined for MET-Gs. (**F**) The overlapping part between prognostic CNV-Gs and MET-Gs. (**G**) Chromosomal localization of the 43 genes and (**H**) their functional annotations. Different colors in the right half circle represent different pathways, the outer ring in the left half circle represents the genes corresponding to the pathway, the corresponding inner ring represents the significant P value, and the connections in the circle represent the relationship between the pathway and genes.

The data of copy number variations, gene expressions and methylations in TCGA were collected to analyze the correlation between CNVs and expression profiles, and between methylations and expression profiles. The correlation distribution between methylation and gene expressions was calculated for all gene promoter regions, and it was found that the overall correlation coefficient was less than 0, suggesting that methylation tended to be negatively correlated with gene expressions. The correlation distribution between gene copy numbers and gene expressions was analyzed, we found that the overall correlation coefficient was greater than 0, suggesting that copy number tended to be positively correlated with gene expressions (Figure 3A). These findings were consistent with previous research. However, a significant difference in the distribution was in the two sets of correlations (D'Agostino test, *p* < 1e-5), suggesting that the overall effect of positive and negative transcriptional dysregulation were caused by abnormal DNA copy number and DNA methylation. A total of 4151 CNV-Gs (Supplementary Table 1) and 2744 MET-Gs (Supplementary Table 2) were identified. The distribution of CNV-Gs and MET-Gs on the genome were analyzed, and we observed that CNV-Gs were mainly distributed on chromosome 12 (Figure 3B), but the MET-Gs were mainly distributed on chromosomes 6 and 7 (Figure 3C). Most of these MET-Gs were protein-coding (Figure 3D), the methylation sites of MET-G were mainly distributed on CpG Island and N shore (2kb area immediately upstream of CpG islands) (Figure 3E), which was consistent with previous studies [20]. The correlations between these genes and overall survival (OS) were examined. Univariate survival analysis determined that 268 CNV-Gs and 125 MET-Gs were significantly related to prognosis of EC (log rank p < 0.05), with an intersection of 43 genes (Figure 3F). These 43 genes were largely distributed on chromosome 7, 12, 20 and 22 (Figure 3G), and were mainly enriched in regulation of protein folding, protein secretion, serine/threonine kinase activity and phosphorylation (Figure 3H). The data suggested that CNVs and methylation might be functionally related to the specifically genes regulated during tumor development.

### Primary identification of molecular subtypes based on CNV-G and MET-G genes

**Figure 4:**
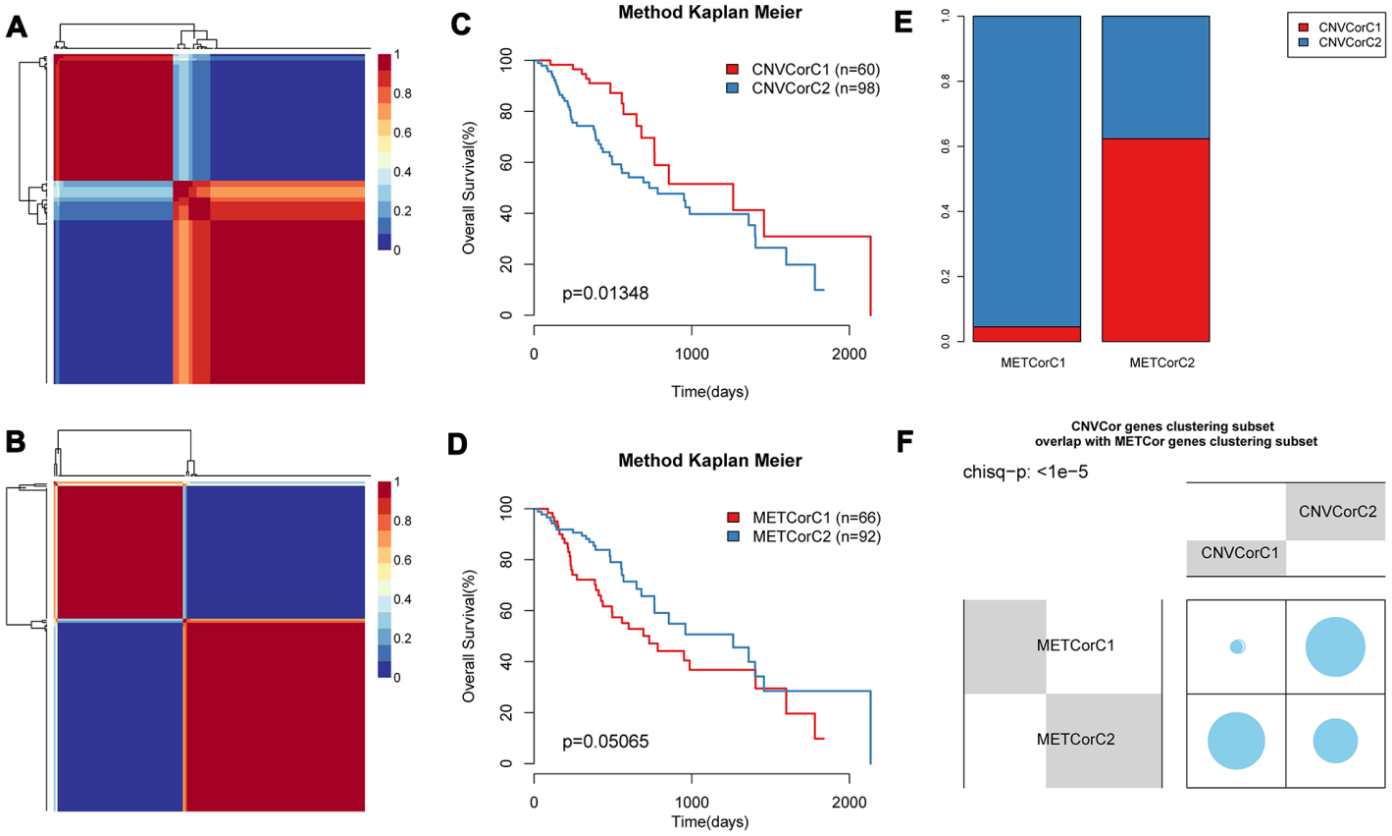
**Identification of molecular subtypes of CNV-G and MET-G genes.** NMF clustering results of (**A**) CNV-Gs and (**B**) MET-Gs were demonstrated, and survival proportions of (**C**) CNV-Gs and (**D**) MET-Gs were shown by Kaplan-Meier curves. (**E**, **F**) The overlapping between the subtypes of CNV-G clustering and the subtypes of MET-G clustering.

Base on NMF, we performed subtyping for CNV-G and MET-G genes and obtained two subtypes (CNVCorC1, N=60 and CNVCorC2, N=98) for the CNV-G gene set (Figure 4A) and two subtypes (METCorC1, N=66 and METCorC2, N=92) for the MET-G gene set (Figure 4B). Significant prognostic differences were identified between CNVCorC1 and CNVCorC2 subtypes (Figure 4C). Although there was no significant difference between METCorC1 and METCorC2, the 3-year survival rate of METCorC2 was significantly better than METCorC1 (Figure 4D). In addition, the subtype relationship between the two molecular types was compared, and a vast majority (96%) of METCorC1 cases belonged to the CNVCorC2 subtype, and 61% of the METCorC2 cases belonged to the CNVCorC1 subtype, with a significant intersection between the two subtypes (Figure 4E, 4F). Such findings were consistent with the relevant regulation of the CNV-G and MET-G genes in EC.

### Multi-omics data based molecular subtyping

**Figure 5:**
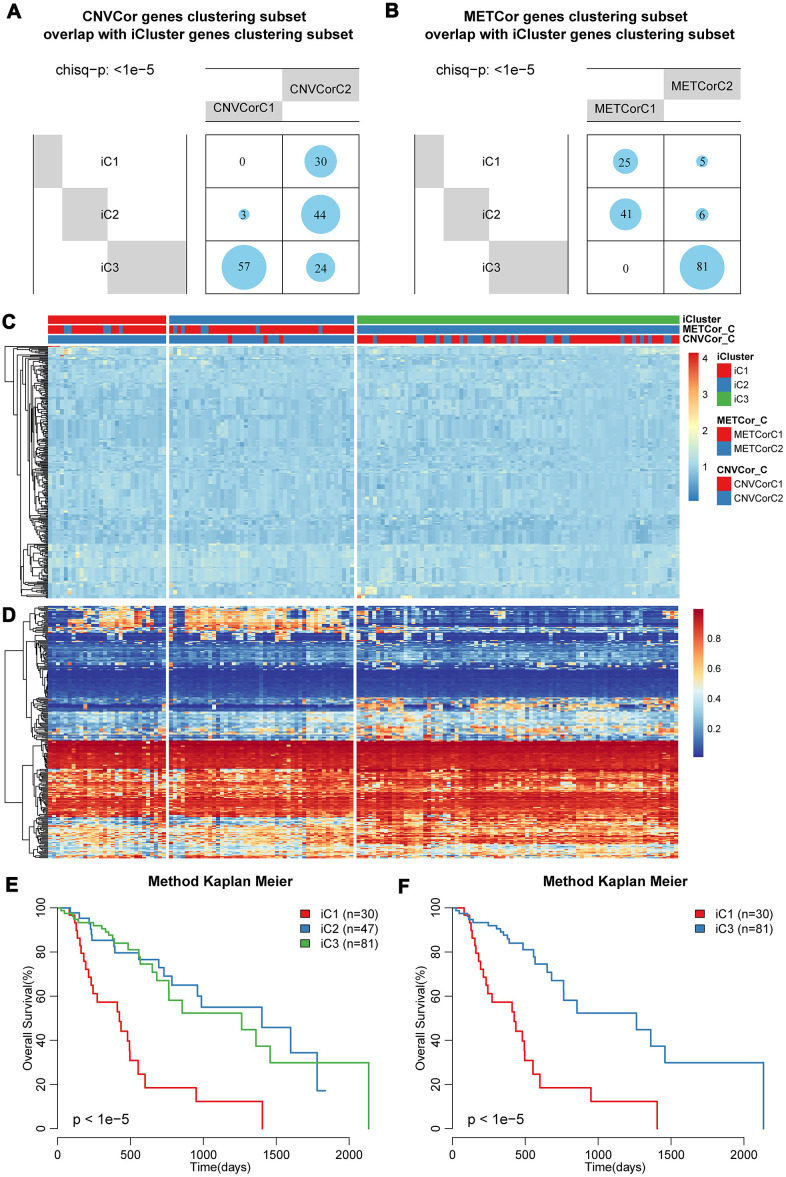
**Identification of molecular subtypes based on multi-omics data.** Overlapping of (**A**) CNV-G or (**B**) MET-G subtypes with iCluster subtypes. The landscape of (**C**) CNV and (**D**) methylation genes across all subtypes. Overall survival proportions for (**E**) each iCluster subtype or (**F**) between iC1 and iC3 subtype.

To further identify molecular subtypes that reflected the multi-layer expression patterns of the CNV-G and MET-G genes, the genomic data of DNA copy numbers, DNA methylations and RNA expressions were integrated using iCluster (an integrated clustering method) with the number of clusters (K) = 2 or 3. The final lambda value of K=2 was 0.26756757, 0.02432432, 0.29459459, and the lambda value of K=3 was 0.95945946, 0.01351351, 0.57567568. To evaluate the optimal clustering results of iCluster, we repeated clustering 20 times at K=2 and K=3, respectively, and found that prognostic diversity of K=2 showed more significant clustering results (Supplementary Figure 1) than the results when K=3 (Supplementary Figure 2). Finally, the patient cohort was aggregated into three subclasses as follows: iC1 (N=30), iC2 (N=47), and iC3 (N=82). These subtypes were consistent with the classification of CNV-G molecular typing and MET-G molecular typing based on NMF analysis, respectively (Figure 5A, 5B) (*p* < 1e-5, χ2 test).

The landscape of CNVs and methylation modes between these three subtypes was shown in Figure 5C, 5D. It should be noted that iC1 had the worst OS among the three subgroups, while iC3 had a significantly better OS (Figure 5E, 5F). Prognostic differences between iC1 and iC2, or between iC2 and iC3 were displayed in Supplementary Figure 3A–3B, it could be found that the disease-free survival of these same subtypes was clearly different (Supplementary Figure 3C). These results indicated that a comprehensive analysis of the CNV-G and MET-G genes facilitated the identification of molecular subtypes, each of which had different combinations of genomic and epigenome features associated with transcriptional disorders and were correlated with different prognosis.

### Clinicopathological and microenvironmental characteristics of molecular subtypes

**Figure 6:**
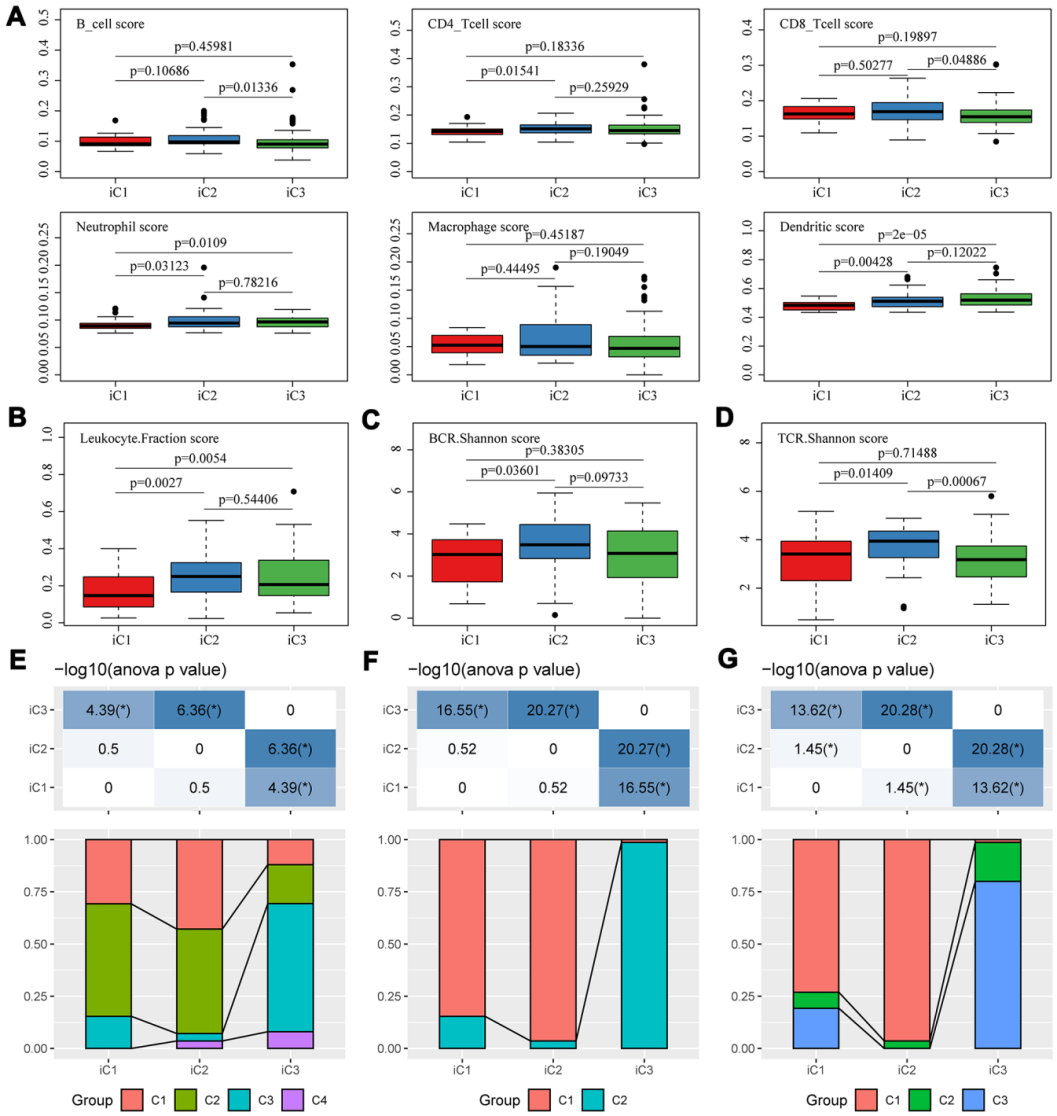
**Microenvironmental characteristics of molecular subtypes.** Distribution of (**A**) six immune cell scores, (**B**) leukocyte fractions, (**C**) BCR Shannon scores, (**D**) TCR Shannon scores across three subtypes. The mutual correlation between subtypes iC1-iC3 and TCGA-EC subtypes C1-C3 from the view of (**E**) CNV, (**F**) methylation and (**G**) gene expression.

Differences in clinical features (TNM, Stage, Gender, and Age) were compared among the three subtypes. Despite that the statistic differences were largely insignificant, the subtype iC1 with the worst prognosis showed a higher proportion of adverse clinical features, such as fewer T0/N0/M0 but more stage III/IV cases. Noticeably, iC1 and iC2 were mainly composed of adenocarcinoma cases, while iC3 were mainly composed of squamous subtypes (Supplementary Figure 4A). We then determined the diversity of tumor immune microenvironment (TIME) score for the three subtypes, and found that iC1 had the lowest stromal score, immune score, and estimate score (Supplementary Figure 4B). Furthermore, the tumor microenvironment of these three subtypes were analyzed and the immune cell content of the three subtypes were compared. We calculated the distribution of six types of immune cell scores for the three subtypes, and observed that iC2 had the highest B cell, CD4+ T cell and CD8+ T cell scores, while iC1 had the lowest Neutrophil/Dendritic scores (Figure 6A). The diversity of tumor immune microenvironment (TIME) score for the three subtypes were calculated, and it could be found that iC1 had the lowest stromal score, immune score, and estimate score (Supplementary Figure 4). The difference in white blood cell ratio and BCR/TCR diversities of the three subtypes were also analyzed, as expected, iC1 had the lowest leukocyte ratio (Figure 6B), while iC2 had the highest BCR and TCR Shannon scores (Figure 6C, 6D). These results suggested that the iC1 subtype was in a state of immunosuppression, which could explain the poor clinical outcome in iC1 subtype compared with other two subtypes.

As molecular subtyping has been proposed by TCGA study of EC [14], we compared the mutual correlation between our subtypes (iC1-iC3) and TCGA-EC subtypes (C1-C3). For CNV, iC1/iC2 were composed of both C1 and C2, while iC3 mainly overlapped with C3 (Figure 6E); for methylation, iC1/iC2 were mainly composed of C1, while iC3 shared a consistency with C2 (Figure 6F); for transcriptional expression, iC1/iC2 were mainly composed of C1, while iC3 was mainly composed of C3 (Figure 6G). Collectively, these similarities and diversities suggested that our subtyping classified based on multi-omics was complementary to the TCGA-EC subtypes.

### Molecular characteristics of three molecular subtypes

**Figure 7:**
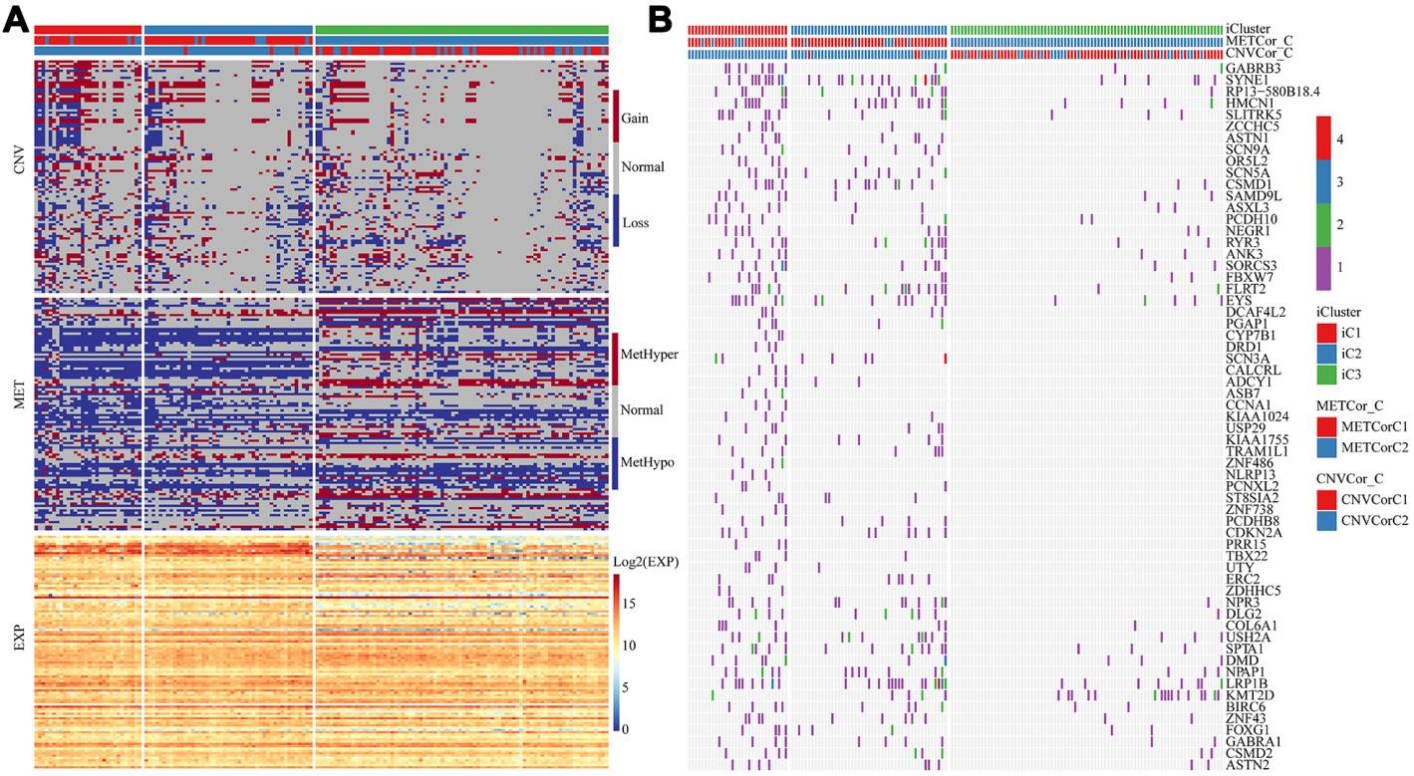
**Multi-omics molecular landscape of the subtypes.** (**A**) Heat map of differential CNV, methylation site and gene expressions across molecular subtypes. (**B**) Heatmap of mutations across molecular subtypes.

To explore the differences in CNV, methylation, and gene expressions between the worst prognostic iC1 and the optimal prognostic iC3 subtype, Fisher-exact test was used to identify the distribution of CNVs (Gain, Loss and Normal) and differences in methylation (HyperMethy, HypoMethy and Normal), and DEseq2 was used to screen differences in gene expressions for the two subtypes. A total of 78 CNV genes (Supplementary Table 3), 285 methylation sites (108 genes, Supplementary Table 4), and 5154 expression genes (Supplementary Table 5) were identified to be significantly diverse between iC1 and iC3 subtypes (Figure 7A). Differences in single nucleotide mutations between subtypes iC1 and iC3 subtypes were also analyzed, and we found 61 genes with significantly higher mutation frequencies in iC1 than in iC3 samples (Figure 7B, Supplementary Table 6). Of the 61 genes, several candidates (such as GABRB3, SYNE1, RP13-580B18.4, HMCN1 and SLITRK5) were related to the development of EC. Specifically, GABRB3 is an inhibitory gene of head and neck cancer [21]; SYNE1 gene hypermethylation can be used as biomarkers in colorectal [22]; SYNE1 polymorphisms are associated with the risk of developing invasive epithelial ovarian cancer [23]; intratumoral heterogeneity of HMCN1 mutant alleles is associated with poor prognosis of breast cancer patients [24]; the combination of SLITRK5 and TP53 is associated with the clinical outcome of gastric cancer patients [25].

**Figure 8:**
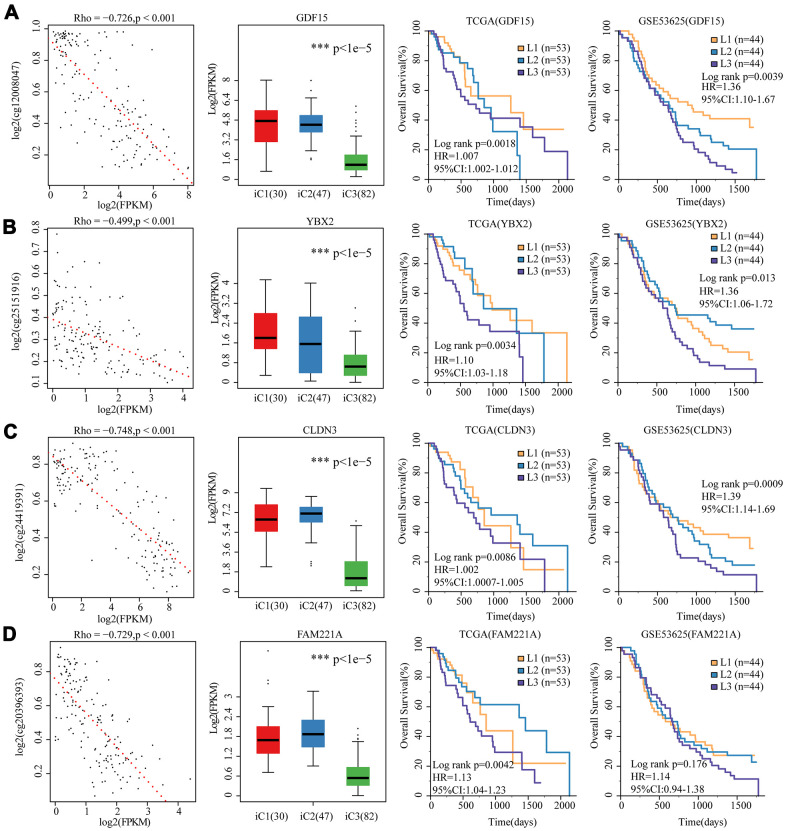
**4 genes as potential biomarkers for the three molecular subtypes.** The relationship between gene expression (horizontal) and methylation (vertical) levels (left panel), expression distribution in three iCluster subtypes (middle panel), and overall survival proportions in TCGA and GSE53625 data sets (right panel) were analyzed for (**A**) GDF15, (**B**) YBX2, (**C**) CLDN3 and (**D**) FAM221A.

To further investigate the relationship among gene expressions, CNVs, and methylation, univariate survival analysis identified a total of 19 differentially expressed genes between iC1 and iC3 subtypes and between CNV gain/loss and hypo/hyper methylation. Four genes (GDF15 (*p*=0.0018), YBX2 (*p*=0.0034), FAM221A (*p*=0.0041), CLDN3 (*p*=0.0087)) were found to be significantly associated with prognosis, all of them were low-expressed in iC3 subtype. We observed in both TCGA-EC and GSE53625 datasets that these 4 genes were adverse prognostic factors, and their elevated expression cascades from low (L1), middle (L2) to high (L3) were consistent with an overall survival lowering from iC1 to iC3 subtypes (Figure 8A–8D). Therefore, these genes might be potential biomarkers of three molecular subtypes.

## DISCUSSION

As a hallmark of malignancy, genomic instability leads to DNA copy number variations in multiple cancer types [26, 27], and these CNVs were important factors affecting changes in gene expressions [28]. In addition to copy number abnormalities, DNA methylation is a critical regulator of gene transcription and one of the most studied epigenetic modifications [29]. Abnormal hypomethylation could induce genomic instability and overexpression of oncogenes, while hypermethylation of the tumor suppressor promoter region disrupts cell cycle regulation, apoptosis and DNA repair, and leads to malignant cell transformation [30]. Recent studies have shown that genomic, epigenomic and transcriptomic dysregulations play crucial roles in the development and progression of tumors [16, 31]. Thus, comprehensively analyzing the multi-layer genomic features of cancer could help identify molecular subtypes, providing new mechanisms and clinical insights into tumor heterogeneity for finding candidate therapeutic targets and biomarkers.

The relationship between genomic, epigenetic and potential regulatory machineries in EC has not yet been investigated. Therefore, we were interested in analyzing the relationship between epigenetic and CNVs using 159 samples from TCGA, and found that DNA copy number abnormalities were consistent with methylation abnormalities. Moreover, we identified CNV-G and MET-G gene sets based on multi-omics association analysis, and established the relationship between CNV and methylation according to gene expressions. Finally, three molecular subtypes (iC1, iC2, iC3) were identified by combining CNV, methylation and gene expression information through multi-omics clustering. Here, iC1 was found to be associated with adverse clinical outcomes, but iC3 was related to favorable clinical outcomes.

Significant differences in the tumor immune microenvironment of the three molecular subtypes were examined. Studies had increasingly shown that tumor infiltrating lymphocytes (TILs) are involved in tumor progression and invasiveness. TILs include various lymphocytes with different activities, and the most common lymphocytes are CD8+ and CD4+ T cells [32]. T lymphocyte infiltration of primary tumors is used to predict clinical outcomes of many cancers, including breast cancer [33], head and neck cancer [34], non-small cell lung cancer [35], colorectal cancer [36], and gastric cancer [37]. As found in our study, to some extent, the heterogeneity of EC might be resulted from the unevenly distributed lymphocyte spectrums across iC1-3 subtypes. On the other hand, the neutrophil and dendritic scores of iC1 with the worst prognosis were significantly lower than those of iC2 and iC3, which was in line with a previous report, in which neutrophil was found to be able to serve as a prognostic marker for patients with locally advanced EC [38]. Similarly, iC1 leukocyte ratio was sharply lower than that of iC2 and iC3, and the iC2 TCR and BCR library diversity was significantly different from iC1 and iC2. In conclusion, the three molecular subtypes displayed diverse tumor immune microenvironmental features, the differences of which might be related to their heterogenic clinical outcomes and therefore were potential targets for immunotherapy of EC.

Furthermore, by comparing the molecular characteristics, 4 representative biomarkers (CLDN3, FAM221A, GDF15 and YBX2) were identified and validated in the three subtypes. These four genes predicted poor prognosis and were all significantly low-expressed in iC3, which was the subtype with a low risk of developing EC. In addition, the expressions of the four genes were negatively correlated with methylation, suggesting that their expressions may be influenced by epigenetic regulation. Among the four genes, CLDN3 and GDF15 were reported to be associated with cancer. CLDNs are transmembrane proteins and major components of the tight junction, changes of which will disrupt the intracellular adhesion and promote malignant transformation [39–41]. Abnormal methylation of CLDN3 has been reported to be associated with the occurrence of ESCC [42]. GDF-15, which is a distal member of the transforming growth factor beta (TGF-beta) superfamily, is widely expressed in a variety of mammalian tissues, and its expression is usually induced in conditions associated with cellular stress. Serum level of GDF-15 is closely related to many diseases, including inflammation, cancer, cardiovascular disease and obesity, thus, GDF-15 could be used as a reliable predictor of disease progression. Fisher OM et al found that plasma and tissue levels of GDF15 are significantly elevated in Barrett's oesophagus and oesophageal adenocarcinoma patients, showing potential in the diagnosis and monitoring of Barrett's disease [43]. These findings supported the application of these genes as biomarkers for identifying EC subtypes.

Although we systemically analyzed the epigenetics, genomics and transcriptomics data of EC in this study, some limitations should be noted. Firstly, with limited clinical follow-up information, we did not consider factors such as the presence of other health status of the patients in affecting clinical outcomes. Secondly, current results were obtained only through bioinformatics analysis and may be biased, thus further genetic and experimental studies involving larger populations should be conducted. Apart from these limitations, our work provided molecular characteristics of EC based on multi-omics.

## CONCLUSIONS

In conclusion, we investigated the molecular characteristics of EC through multi-omics analysis of genomics, epigenomics, and transcriptomics data. We found that CNV and methylation of DNA play important roles in EC, and identified three potential clinically relevant molecular subtypes and four key biomarkers. These novel classifications may facilitate the development of precision medicine for treating EC patients.

## MATERIALS AND METHODS

### Data origination

The Cancer Genome Atlas (TCGA) (https://portal.gdc.cancer.gov/) dataset for EC was downloaded with GDC API (https://gdc.cancer.gov/developers/gdc-application-programming-interface-api). Here, we obtained 185 samples with CNV detection, 168 samples with methylation (MET) data, 195 samples with RNA-seq detection and 184 samples with SNP data. A total of 159 primary tumor samples with CNV, methylation, RNA-seq, and SNP data were selected, and the clinical follow-up information of these 159 samples (Supplementary Table 7) were downloaded. Another EC dataset, GSE53625 (https://www.ncbi.nlm.nih.gov/geo/query/acc.cgi?acc=GSE53625) [44], was downloaded from Gene Expression Omnibus (GEO) database (https://www.ncbi.nlm.nih.gov/geo). The data platform was agilent-038314 CBC Homo sapiens lncRNA + mRNA microarray V2.0 (Feature Number version), which contained a total of 358 samples incorporating 179 EC samples and 179 normal samples. Data of all samples were shown in Table 1.

**Table 1:** Demographic and clinical characteristic descriptions for esophageal carcinoma patients in different datasets.

**Characteristics**	**TCGA**	**GSE53625**
Number of samples	159	179
Median survival time (95%CI) (Days)	536 (468-605)	1088 (986-1189)
Number of death (%)	63 (39.6)	106 (59)
Age (years)	62.3 (±12.1)	59 (±9)
Histology type (%)		
Esophagus adenocarcinoma	79 (49.6)	-
Esophagus squamous cell carcinoma	80 (50.4)	-
unknown	-	179 (100)
Stage_T (%)		
T1	25 (15.7)	12 (6.7)
T2	40 (25.2)	27 (15.1)
T3	87 (54.7)	110 (61.5)
T4	5 (3.1)	30 (16.8)
TX	2 (1.3)	0 (0)
Stage_N (%)		
N0	64 (40.3)	83 (46.4)
N1	69 (43.4)	62 (34.6)
N2	9 (5.7)	22 (12.3)
N3	5 (3.1)	12 (6.7)
NX	12 (7.5)	0 (0)
Stage_M (%)		
M0	126 (79.2)	0 (0)
M1	15 (9.4)	0 (0)
MX	15 (9.4)	0 (0)
unknow	2 (2)	179 (100)
Stage (%)		
Stage I	16 (10)	10 (5.6)
Stage II	71 (44.7)	77 (43)
Stage III	54 (34)	92 (51.4)
Stage IV	14 (8.8)	0 (0)
unknow	4 (2.5)	0 (0)

### Data preprocessing

The CNV data were preprocessed. For the combination of CNV probes, 50% regional overlap in the two intervals was considered the same, while the number of coverage probes < 5 intervals were removed. CNV probe were mapped into the corresponding gene using gtf of the GENCODE [45] GRCh38.p12 version, while multiple CNV probes in one gene region were combined as one, and the combined CNV values were averaged. For preprocessing of methylation data, sites missing from more than 70% of samples were removed. The missing values were filled by the k-Nearest Neighbour (KNN) algorithm [46], and the gtf upstream of the TSS and the downstream 200 bp CpG probe were retained using the gtf of the GENCODE GRCh38.p12 version and mapped into the corresponding gene. For RNA-seq data, low-expressed genes in each sample (the sample with fragments per kilobase of transcript per million mapped reads (FPKM) of 0 accounted for < 0.5 of the total sample ratio) were removed, while the gene set with higher expression were retained. For SNP data, the file in MAF format was parsed, the mutations in the intron interval and the mutations annotated as silence were removed. For chip data, the standardized expression profile (EXP) matrix was directly downloaded, and probes were then matched to genes according to the annotation information of the platform. The median level of multiple probes matched to the same gene was determined as the expression of the gene, while probes matching to multiple genes were removed.

### Identification of CNV-G gene set and MET-G gene set

The Pearson correlation coefficient (r) of each gene corresponding to CNV and expression profile (RNA-seq), methylation and expression profile were calculated respectively, and the correlation coefficient was converted to z-value according to the formula ln((1+r)/(1-r)). The genes of *p* < 1e-5 with correlation coefficient test constituted a gene set significantly related to CNV (copy number variation genes, CNV-Gs) and a gene set related to methylation (methylation genes, MET-G).

### Identification of molecular subtypes based on single omics data

Nonnegative matrix factorization (NMF) is an unsupervised clustering method widely used in discovering genomics-based tumor molecular subtypes [47, 48]. To further examine the relationship between the expressions of the CNV-G/MET-G gene sets and phenotypes, the samples were clustered by the NMF method based on the expression profiles of the CNV-G and MET-G gene sets, respectively. Then the clinical features of the clustered sample and the link between the molecular subtypes of the two were analyzed, and 50 iterations were performed with the standard "brunet" of the NMF method. The number of clusters K was set to 2-10, then the average profile width of the common member matrix was calculated using the R package NMF [49], with the minimum member of each subclass set as 10. According to the cophenetic correlation coefficient (CPCC), the optimal cluster number for molecular subgroups was determined by dispersion and silhouette indexes based on CNV-G and molecular subgroups based on MET-G.

### Identifying molecular subtypes by multi-omics clustering

[50] The “iCluster” [49] method in the R package was applied to perform multi-group data integration cluster analysis. We first extracted the methylation profile, SNV and gene expression profile data of CNV-G and MET-G as input data, and set these data distributions as Gaussian distributions. To optimize CNV, MET and EXP data weight values (lambda values), 20 iterations were used and 101 lambda sample points were selected between 0-1 for optimal lambda value screening. Cluster analysis with clusters K=2, 3, and 4 was performed to determine the optimal number of clusters, and 20 iterations were repeated at each cluster to analyze the cluster stability. Finally, molecular subgroups with stable clusters were obtained.

### Assessing the relationship between molecular subtypes and tumor microenvironment

[51] TIMER [51] is a web resource for systematical evaluations of the clinical impact of different immune cells on cancers, including the evaluation of the abundance of six immune cell types B cell, CD4 T cell, CD8 T cell, neutrophil, macrophage and dendritic cell in the tumor microenvironment of TCGA samples. These related data were downloaded, and the abundance distribution of the six types of immune cells corresponding to samples of different molecular subtypes was analyzed, also, statistical differences in the abundance of immune cells of different subtypes were assessed by the rank sum test.

### Analysis of genetic differences in molecular subtypes

DESeq2 [52] is a widely used differential analysis method in transcriptome. Variance-mean dependence in count data was evaluated from high-throughput sequencing assays and test for differential expression based on a model using the negative binomial distribution. DESeq2 [52] was used to examine differences in gene expressions between different molecular subtypes, and 2 fold of the difference plus FDR < 0.05 was selected as a threshold to identify differentially expressed genes between molecular subtypes.

### Relationship between molecular subtypes and tumor genomic variation

To determine the differences in genomic variation between molecular subtypes, SNP data of TCGA-EC were analyzed. Intron and silent mutations were removed, and fisher's exact test was used to analyze the differentially expressed genes between two groups. Gene with a threshold variation of *p* < 0.05 was selected to identify mutational differences.

### Functional enrichment analyses

To analyze the function of the gene set, we used R package clusterprofiler [53] and performed Gene Ontology (GO) analysis to identify over-represented GO terms in three categories (biological processes, molecular function and cellular component). Also, pathway enrichment analysis was conducted referring to the Kyoto Encyclopedia of Genes and Genomes (KEGG) database. A FDR< 0.05 was considered to denote a statistical significance.

### Identification of subgroup-associated prognostic markers

To identify prognosis-related key molecules, differences in CNV, methylation, and gene expression were compared between the subtypes with the worst and optimal prognosis, and genes with abnormalities in different histologies were screened. Furthermore, the prognostic relevance of these genes was analyzed by univariate survival. Finally, prognostic-related gene markers were obtained.

### Survival analysis

By using the R package survival, the prognostic differences between subtypes were visualized through univariate Kaplan-Meier (KM) survival analysis and Log-rank test. *P* < 0.05 was defined as statistically significant. Correlation coefficients greater than 0 and p<0.01 were defined as significant positive correlations, and correlation coefficients less than 0 and p<0.01 were defined as significant negative correlations. All of these analyses were performed in R 3.4.3.

## Supplementary Material

Supplementary Figures

Supplementary Table 1

Supplementary Table 2

Supplementary Table 3

Supplementary Table 4

Supplementary Table 5

Supplementary Table 6

Supplementary Table 7
