# Supplementary material for: Integrative analysis of genomic, epigenomic and transcriptomic data identified molecular subtypes of esophageal carcinoma
Source: Aging (Albany NY). 2021 Feb 26;13(5):6999–7019. doi: 10.18632/aging.202556 (PMC7993659; doi:10.18632/aging.202556)
Supplement: Supplementary Figures [file aging-13-202556-s001.pdf]

SUPPLEMENTARY FIGURES

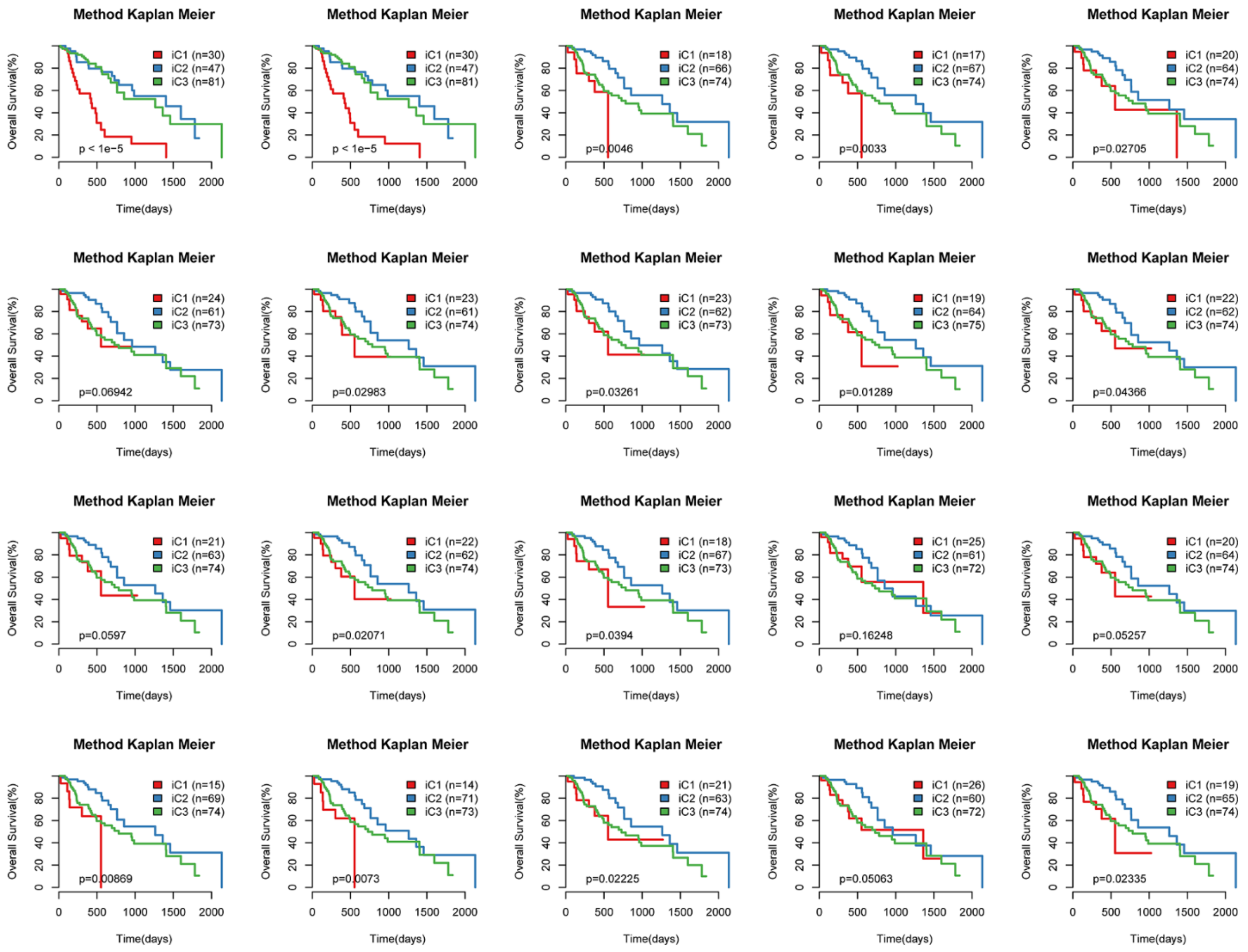

Supplementary Figure 1. Prognostic differences for each of the 20 clustering results identified by iCluster at k=2.

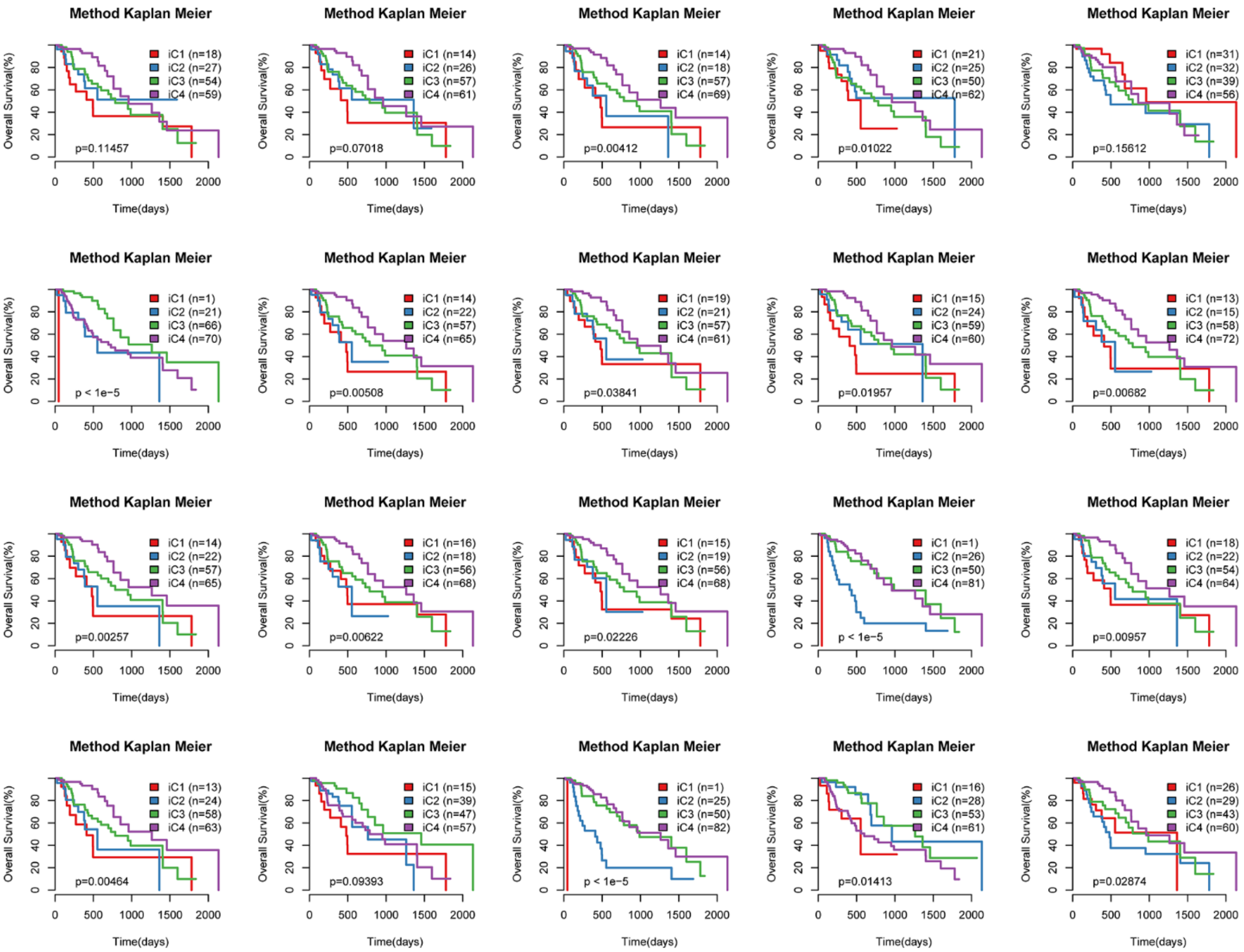

Supplementary Figure 2. Prognostic differences for each of the 20 clustering results identified by iCluster at k=3.

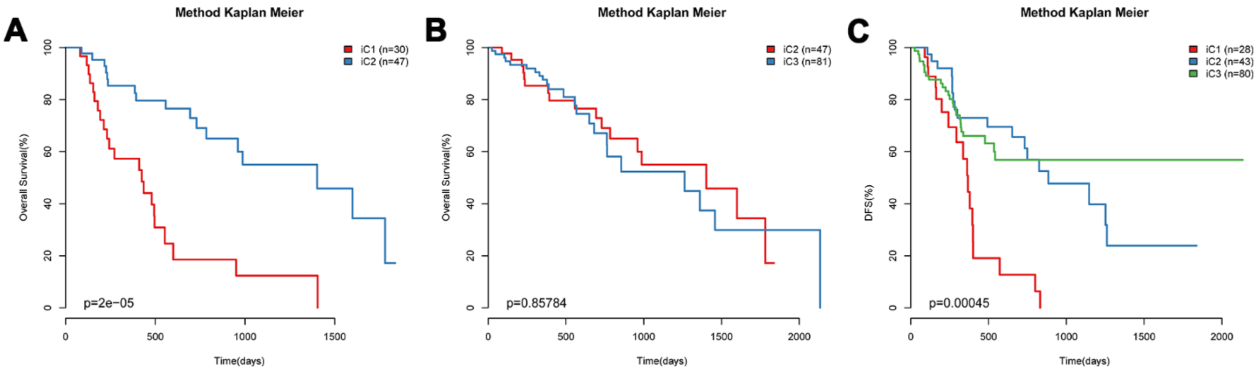

Supplementary Figure 3. Prognostic diversities of the three iCluster subtypes. (**A**) Overall survival diversity between iC1 and iC2. (**B**) Overall survival diversity between iC2 and iC3. (**C**) Progression-free survival proportions for the iC1, iC2 and iC3 subtypes.

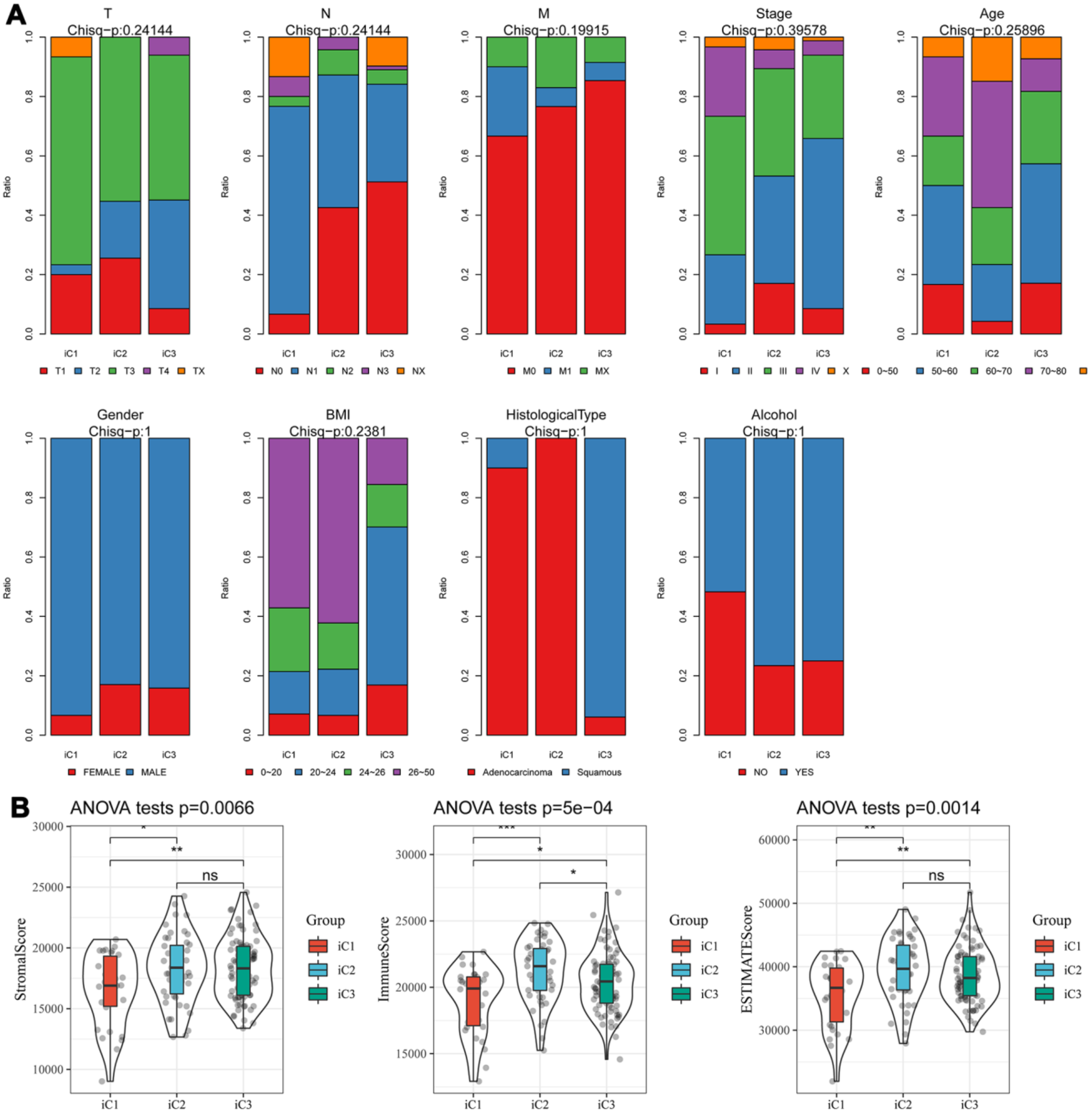

Supplementary Figure 4. Differences in tumor immune microenvironmental and clinical features for three subtypes. (**A**) Distribution of TNM, Stage, Gender, Age, BMI, Histological Type, Alcohol intake in the three subtypes. (**B**) Diversity of tumor immune microenvironment (TIME) score for the three subtypes.
